# Transcriptional profiling and functional characterization of the *Hc-NHR-49* gene in ivermectin resistance of *Haemonchus contortus*

**DOI:** 10.1186/s13071-026-07433-x

**Published:** 2026-05-23

**Authors:** Zeshuang Li, Penglong Wang, Xiaoping Luo, Gaowa Gong, Bin Li, Dandan Liu, Yaning Li, Jiuru Huangfu, Luyang Tang, Xuesen Zhang, Wei Zhang, Junyan Li

**Affiliations:** 1 College of Veterinary Medicine, Xinjiang Agricultural University, No. 311 Nongda East Road, Saybag District, Urumqi, 830052 China; 2 Key Laboratory of Grass-Feeding Livestock Healthy Breeding and Livestock Product Quality Control, Inner Mongolia Academy of Agricultural and Animal Husbandry Sciences, Veterinary Research Institute, No. 22 Zhao Jun Road, Yu Quan District, Hohhot, 010031 China

**Keywords:** *Haemonchus contortus*, Nuclear hormone receptors, Ivermectin, Resistance

## Abstract

**Background:**

The barber’s pole worm (BPW), *Haemonchus contortus*, poses a significant threat to sheep health and livestock husbandry. Control has historically relied on synthetic anthelmintics such as ivermectin; however, widespread resistance to this drug has emerged. The functional role of nuclear hormone receptors (NHRs) in *H. contortus* remains poorly understood.

**Methods:**

The *Hc-NHR-49* gene was polymerase chain reaction (PCR)-amplified and subjected to bioinformatic analysis. Expression profiles of the gene in resistant and sensitive strains across different developmental stages and under ivermectin stress were examined using Quantitative reverse transcription PCR (RT‑qPCR). Polyclonal antibodies were generated in mice via recombinant prokaryotic expression and validated by Western blot. The spatial expression pattern of Hc‑NHR‑49 was further determined by immunohistochemistry. Finally, RNA interference followed by larval head swing assays was performed to assess its functional role in ivermectin response.

**Results:**

In this study, we cloned and characterized the nuclear hormone receptor gene Hc-NHR-49 from *H. contortus*. The full-length complementary DNA (cDNA) is 1272 bp, encoding a 423-amino-acid protein. Bioinformatics analysis revealed that Hc-NHR-49 is highly conserved across species, suggesting functional similarity. Recombinant Hc-NHR-49 was expressed and purified. Polyclonal antibodies raised in mice specifically recognized native Hc-NHR-49 in somatic extracts, as confirmed by Western blot. Immunohistochemical localization showed that Hc-NHR-49 is widely distributed, with particularly high expression in the intestine, uterus, ovaries, and testes. Transcript levels were detected throughout all developmental stages in both ivermectin-susceptible and -resistant strains. To investigate its association with ivermectin resistance, worms were exposed to the half maximal effective concentration (EC_50_) of ivermectin, which elicited a plastic expression response of *Hc-NHR-49*. RNA interference (RNAi)-mediated knockdown of Hc-NHR-49 increased ivermectin susceptibility in resistant parasites.

**Conclusions:**

Collectively, our results suggest that *Hc-NHR-49* is implicated in ivermectin resistance in *H. contortus*. These findings contribute to a deeper understanding of resistance mechanisms and could inform the future development of alternative control measures.

**Graphical Abstract:**

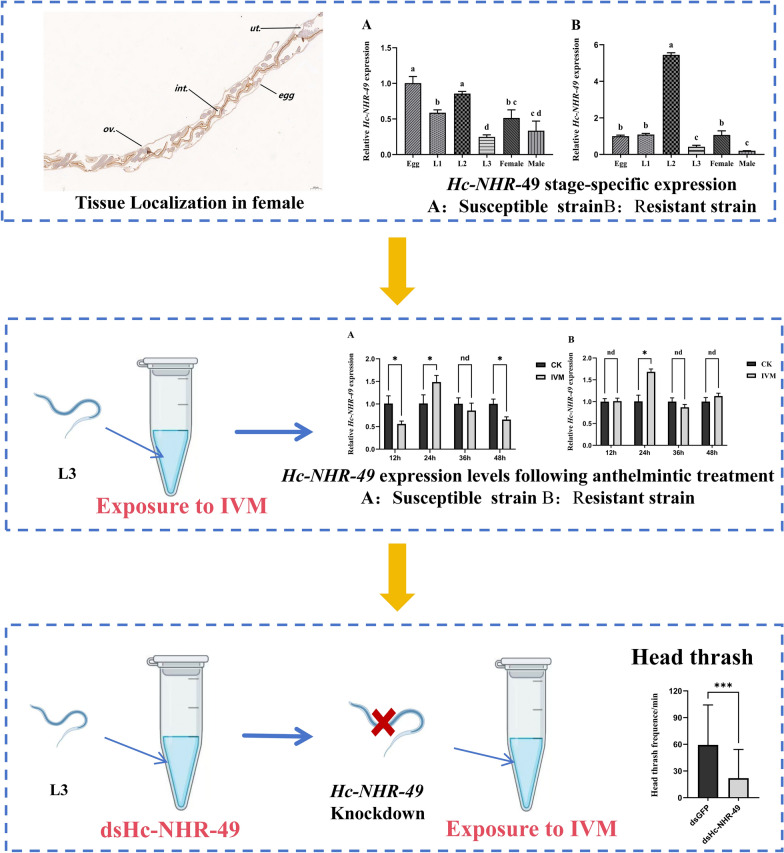

**Supplementary Information:**

The online version contains supplementary material available at 10.1186/s13071-026-07433-x.

## Background

*Haemonchus contortus*, a hematophagous gastrointestinal nematode, parasitizes the abomasum of ruminants and is responsible for substantial global economic losses in livestock production [1]. The extensive and often indiscriminate use of anthelmintics has inevitably led to the widespread emergence of drug resistance worldwide, severely compromising the efficacy of chemical control against nematodes. Anthelmintic resistance (AR) to *H. contortus* has been extensively documented. For instance, a survey across 46 farms in eight southern US states revealed that populations of *H. contortus* exhibited resistance to benzimidazole, levamisole, ivermectin (IVM) and moxidectin on 98, 54, 76 and 24% of the farms, respectively [2]. In northern Brazil, fecal egg count reduction tests were conducted on ten farms, which indicated that 37.1% of the pastures harbored nematode populations resistant to ivermectin [3]. Larval development assays demonstrated a high prevalence of resistance in gastrointestinal nematodes (*H. contortus* and *Trichostrongylus colubriformis*) from Colombian goats and sheep to albendazole, ivermectin, moxidectin, and levamisole [4]. In eastern Inner Mongolia, China, a study of ten grazing sheep farms reported an average gastrointestinal nematode infection rate of 79.2% (range: 45–100%) and a mean fecal egg count of 1813 eggs per gram (range: 0–32,400), with evidence of varying levels of resistance to avermectin, ivermectin, and albendazole [5]. Despite the significant global threat posed by anthelmintic resistance in gastrointestinal nematodes, the precise molecular and genetic mechanisms driving this phenomenon remain incompletely understood.

Recent research has established that enhanced enzymatic detoxification and target-site insensitivity are the principal mechanisms underlying anthelmintic resistance in nematodes [6]. Among the various documented pathways, elevated levels of anthelmintic resistance were frequently correlated with the activity of metabolic detoxification enzymes. P-glycoproteins (*P-gps*), belonging to the ATP-binding cassette (ABC) transporter family, function as efflux pumps that expel hydrophobic xenobiotics from cells, thereby reducing intracellular concentrations of anthelmintics [7, 8]. For example, *Caenorhabditis elegans* with knocked-out *P-gps* showed increased sensitivity to IVM following anthelmintic exposure, confirming the involvement of these transporters in resistance [9]. In *H. contortus*, strains that survive under IVM pressure exhibit upregulation of *P-gp-2* and *P-gp-9* genes [10]. Similarly, previous studies have associated glutathione S-transferase (GST) with resistance [11]. Inhibition of GST activity in *H. contortus* resulted in a significant reduction in survival rate of third-stage larvae (L3s), ultimately increasing susceptibility to thiabendazole [12, 13]. While numerous studies have linked detoxification enzymes to altered ivermectin sensitivity in nematodes, the specific transcription factors that regulate the expression of these genes remain largely uncharacterized.

Nuclear hormone receptors (NHRs), members of the nuclear receptor superfamily, function as transcription factors that regulate signaling pathways and gene expression by binding lipophilic ligands [14]. They play a vital role in diverse biological processes, including xenobiotic metabolism, development and reproduction [15]. In *H. contortus*, a blood-feeding nematode, 40 NHR genes were found to respond to host serum in vitro, with one (*Hc-NHR-64*) consistently activated by anthelmintic exposure [16]. A high-throughput RNA interference (RNAi) screen targeted 43 NHR genes in *H. contortus* and identified at least two essential genes: *Hc-NHR-105*, crucial for viability, and *Hc-NHR-17*, necessary for larval development in vitro [16]. Recent studies have indicated that *Ce-NHR-8* in *C. elegans* regulates ivermectin resistance by upregulating IVM detoxification genes. Similarly, RNAi silencing of *Hc-NHR-8* in larvae increased IVM sensitivity in both susceptible and resistant strains of *H. contortus*, suggesting functional conservation between *Hc-NHR-8* and *Ce-NHR-8* [17]. Furthermore, *Hc-NHR-49*, a regulator of lipid metabolism and the oxidative stress response in nematodes, has also been linked to ivermectin resistance. Genome-wide association studies have identified *Hc-NHR-49* as a candidate gene associated with IVM resistance in *H. contortus* [18, 19]. However, this gene has not yet been fully characterized in the parasite, and the relationship between its expression dynamics and the progression of resistance remains unclear.

Therefore, the identification of *Hc-NHR-49* in *H. contortus* will elucidate of ivermectin resistance in this species and may inform the development of targeted control strategies. Using a previously established transcriptomic database for *H. contortus*, we identified the *Hc-NHR-49* gene by querying the database with NHRs search terms. This study presents the cloning, recombinant expression, and immunohistochemical staining of *Hc-NHR-49*. Moreover, to investigate its role in anthelmintic resistance, we analyzed the transcriptional response of *Hc-NHR-49* to ivermectin exposure and employed RNAi to knockdown its messenger RNA (mRNA) in an ivermectin-resistant strain.

## Methods

### Parasites

The susceptible population of *H. contortus* (YCHc-022) was originally collected in 2018 from a farm in the Yuci District of Shanxi, China. The ivermectin-resistant population (CYHHc-136) was collected in the same year from a farm in the Ulanqab District of Inner Mongolia, China. These populations have been maintained in the laboratory for over 6 years without exposure to any anthelmintic. The ivermectin-resistance status of these populations was confirmed through the fecal egg count reduction test, performed as previously described [20].

### Sample collection

In preparation for *H. contortus* infection, rectal fecal samples were collected from sheep, and the McMaster technique was used to confirm that all sheep were free of nematode eggs. After a 1-week adaptation period, sheep were divided into groups and orally inoculated with 5000 infectious third-stage larvae (L3). One group received L3 from a susceptible strain, and the other received L3 from a resistant strain. Approximately 21 days postinfection, fecal samples from the infected sheep were collected and incubated at 27 ℃ for 7 days to allow larval development to L3. The L3 larvae were then collected using a Baermann apparatus [21], thoroughly washed with deionized water, and stored at 16 ℃ until use.

The egg collection process involved the following steps: Fresh fecal samples were homogenized on the day of collection and mixed with saturated saline solution. The mixture was sequentially filtered through sieves with mesh sizes of 20, 40, 100, 200 and 300. Nematode eggs were then recovered using the saturated saline flotation method. Subsequently, the collected liquid was filtered through a 500-mesh sieve, followed by rinsing the sieve with physiological saline to collect the wash solution into a beaker. After allowing the wash solution to settle for 20 min, the supernatant was discarded, and the egg suspension was transferred to a 15 mL centrifuge tube. The suspension was left to stand at room temperature for 15 min, after which the supernatant was discarded. This washing step was repeated until the eggs were thoroughly cleaned.

To prepare the larval culture, distilled water (1–2 mL) was added to each well of a 6-well plate, followed by an appropriate amount of nematode eggs. After incubation at 27 °C for 36 h, first-stage larvae (L1) were collected. The remaining cultures were further incubated at 27 °C for 3 days, after which second-stage larvae (L2) were collected and stored at −80 °C until use. L1 and L2 larval stages were distinguished by microscopic examination on the basis of differences in body length and motility [22, 23].

In regard to adult collection, according to the requirements of the Animal Welfare Regulations of the Inner Mongolia Academy of Agricultural and Animal Husbandry Sciences, sheep infected with a susceptible strain (YCHc-022) and a resistant strain (CYHHc-136) were dissected. The abomasum was excised, and its contents were emptied into an enamel pan followed by the addition of an appropriate volume of physiological saline. Fresh adult parasites were carefully extracted using a fine needle. Female and male worms were identified macroscopically. *H. contortus* exhibits typical morphological characteristics, with females displaying a distinctive red-and-white twisted (“barber pole”) appearance [24]. Following rinsing with distilled water, the collected parasites were promptly preserved in liquid nitrogen or 4% paraformaldehyde for subsequent use.

### Total RNA extraction and reverse transcription

Total RNA was extracted from ten adult female nematodes of the YCHc-022 strain for amplification of full-length *Hc-NHR-49* cDNA. Briefly, nematodes were homogenized in TRIzol Reagent (15596026, invitrogen, USA) using an OSE-Y20 tissue homogenizer (Tiangen Biotech, China), following the manufacturer’s protocols. The extracted RNA was dissolved in 30 μL of diethylpyrocarbonate (DEPC)-treated water, quantified by measuring the absorbance at 260 nm, and assessed for purity using the A260/A280 ratio. Reverse transcription was performed with the TransScript One-step gDNA Remover and cDNA Synthesis SuperMix (R323, Vazyme Biotech, China). The resulting cDNA was stored at −20 °C for subsequent use.

### Cloning of *Hc-NHR-49* gene and sequence analysis

The full-length *Hc-NHR-49* cDNA was amplified by PCR using gene-specific primers (Table S1), which were designed from our in-house *H. contortus* transcriptome database. The PCR conditions were as follows: initial denaturation at 94 °C for 5 min; 35 cycles of 94 °C for 15 s, 50 °C for 15 s, and 68 °C for 30 s. The PCR products were purified with the TIANgel Midi Purification Kit (TIANGEN, China) and cloned into the pEASY^®^-Blunt Cloning Vector (TransGen, China) according to the manufacturer’s protocols. Conserved protein domains were identified by multiple amino acid sequence alignment using Clustal Omega. For phylogenetic analysis, the nucleotide sequence of *Hc-NHR-49* was aligned with homologous sequences from other species retrieved from GenBank, and a phylogenetic tree was constructed in MEGA 11 using the neighbor-joining method with 1000 bootstrap replicates. The *Hc-NHR-49* sequence has been deposited in GenBank under accession number PX701906.

## Transcriptional analysis of *Hc-NHR-49* in *H. contortus*

### Analysis of *Hc-NHR-49* expression in different development stages

To examine the stage- and strain-specific expression of *Hc-NHR-49*, samples were collected from both ivermectin-susceptible (YCHc-022) and -resistant strains (CYHHc-136) of *H. contortus*. The developmental stages analyzed included adult females, adult males, larvae, and eggs. For each biological replicate, the sample consisted of 10 adults (male or female), 5000 larvae, or 10,000 eggs per strain. This sampling was performed in triplicate. Total RNA was then extracted from each sample (20 μL) and reverse transcribed (1 μg RNA) for cDNA synthesis. The transcriptional levels of *Hc-NHR-49* across different stages were quantified by RT-qPCR, using eggs as the reference control, as described in Section 2.6.

### Ivermectin-induced expression of the *Hc-NHR-49* gene

To evaluate the expression of *Hc-NHR-49* in response to ivermectin in both resistant and susceptible strains, approximately 5000 L3 larvae per strain were collected. They were then exposed separately to either ivermectin at their respective EC_50_ concentrations or to 0.5% dimethyl sulfoxide (DMSO) (vehicle control). Larvae were harvested at 12, 24, 36, and 48 h postexposure and transferred to 1.5 mL centrifuge tubes. Each treatment was performed in triplicate. Finally, the transcript levels of *Hc-NHR-49* in the collected samples were quantified by RT-qPCR as described in Section 2.6.

### Real-time quantitative PCR

The expression of *Hc-NHR-49* across different life stages and strains and after ivermectin induction was quantified by RT-qPCR using a CFX96^™^ Real-Time PCR System (Applied Biosystems). Gene-specific primers were designed with Primer 5.0 (Table S1). Each 20 μL reaction contained 10 μL of TransStart Tip Green qPCR Super Mix, 7.2 μL of double-distilled (ddH_2_O), 0.4 μL each of forward and reverse primers (10 μM), and 2 μL of cDNA template. The thermal cycling protocol consisted of an initial denaturation at 95 °C for 30 s, followed by 40 cycles of 95 °C for 10 s and 60 °C for 30 s. Fluorescence was acquired at the end of the 60 °C extension step. A melt curve analysis (60 °C to 95 °C) was performed to confirm amplicon specificity. Three independent biological replicates were analyzed for each sample, with *GAPDH* used as the endogenous control gene [25]. Relative gene expression was calculated using the 2^−ΔΔCT^ method [26].

### Expression and purification of the recombinant *Hc-NHR-49*

The *Hc-NHR-49* gene was synthesized and cloned into the pCold-TF expression vector via *XhoI* and *Hindlll* restriction sites (Table S1). The resulting recombinant plasmid, pCold-TF-*Hc-NHR-49*, was transformed into *Escherichia coli* BL21 (DE3) competent cells. Protein expression was induced by adding 0.5 mM isopropyl β-D-1-thiogalactopyranoside (IPTG) and incubating at 16 °C for 24 h. Cells were then harvested, lysed, and centrifuged. The soluble recombinant *Hc-NHR-49* (rHc-NHR-49) protein was purified from the supernatant using Ni–NTA Agarose (AS045, ABclonal, China) according to the manufacturer’s protocols. The purified protein was analyzed by sodium dodecyl sulfate polyacrylamide gel electrophoresis (SDS-PAGE), quantified with a BCA protein assay kit (PC0020, Solarbio, China), aliquoted and stored at −80 °C for subsequent use.

### Polyclonal antibodies and Western blot analysis

Mouse antiserum was obtained as follows: For the initial immunization, 100 μg of the purified recombinant protein was emulsified with an equal volume of Freund’s complete adjuvant and administered subcutaneously to 7-week-old Kunming mice. A secondary immunization was given 14 days later, consisting of 50 μg of the recombinant protein emulsified with an equal volume of Freund’s incomplete adjuvant. After an additional 14 days, a third booster immunization was performed following the same protocol as the second immunization. Serum was collected via orbital sinus bleeding 7 days after the final booster, centrifuged, and the supernatant stored at −20 °C for subsequent use.

The reactivity and specificity of the mouse antiserum were analyzed by Western blot. Samples containing 50 ng of purified rHc-NHR-49 or 10 μg of soluble nematode extract were resolved by SDS-PAGE and transferred onto polyvinylidene difluoride (PVDF) membranes. The membranes were blocked with 5% [weight/volume (w/v)] skimmed milk in phosphate-buffered saline (PBS) with Tween^®^−20 (PBST) (PBS with 0.1% Tween-20) for 1 h at room temperature. They were then incubated with the primary mouse antiserum at a 1:500 dilution in blocking buffer for 1 h. After washing, the membranes were incubated with a horseradish peroxidase (HRP)-conjugated goat anti-mouse IgG secondary antibody (HS201, TransGen, China) at a 1:10,000 dilution for 1 h. Following additional washes, immunoreactive bands were detected using an enhanced chemiluminescence (ECL) substrate (180–5001, Tanon, China) according to the manufacturer’s instructions.

### Immunohistochemical localization of *Hc-NHR-49* in *H. contortus* tissues

Fresh adult female and male nematodes were fixed in 4% paraformaldehyde and embedded in paraffin. Tissue sections were deparaffinized in xylene and rehydrated through a graded ethanol series. Antigen retrieval was performed by heating the sections in citrate buffer (pH 6.0). Subsequently, endogenous peroxidase activity was quenched by incubation in 3% hydrogen peroxide at room temperature for 25 min in the dark, followed by three 5-min washes in PBS (pH 7.4). Nonspecific binding was blocked with 3% bovine serum albumin (BSA) for 30 min at room temperature. After blocking, sections were incubated overnight at 4 °C with the primary antibody diluted 1:800 in PBS within a humidified chamber. Following three 5-min washes in PBS, sections were incubated for 50 min at room temperature with HRP-conjugated goat anti-mouse secondary antibody (1:5000). After another three washes in PBS, immunoreactivity was visualized by applying freshly prepared 3,3′-diaminobenzidine (DAB) chromogen to the sections. The DAB reaction was monitored under a light microscope and stopped when optimal signal-to-noise contrast was achieved, with positive staining appearing as a brown-yellow precipitate. Sections were then counterstained, dehydrated, cleared, and mounted for microscopic examination.

### RNAi in nematodes

Regarding larval preparation and exsheathment, active third-stage larvae of the ivermectin-resistant (CYHHc-136) strains were used. Approximately 5000 L3 were exsheathed by incubation in 0.6% NaClO at 36 °C for 50 min with periodic gentle inversion. Complete exsheathment was confirmed by microscopic examination. The exsheathed larvae were then washed three times with 0.9% NaCl solution, followed by three rinses in diethylpyrocarbonate (DEPC)-treated water.

In regard to production of double-stranded RNA (dsRNA) in *E. coli*, double-stranded RNAs (dsRNAs) targeting the gene of interest (dsHc-NHR-49) and green fluorescent protein (GFP; control) were expressed using the L4440 vector system. The L4440 plasmid contains two convergent T7 promoters flanking a multiple cloning site and an ampicillin resistance marker [27]. Target fragments were amplified by PCR with gene-specific primers (Table S1) and engineered with *XhoI* and *NotI* sites for directional cloning into L4440. The resulting recombinant plasmids (L4440-dsHc-NHR-49 and L4440-dsGFP) were verified by sequencing.

Regarding expression and extraction, the verified plasmids were independently transformed into the RNase III-deficient *E. coli* HT115 (DE3) strain, which is incapable of degrading dsRNA. Positive transformants were selected on Luria–Bertani (LB) agar plates containing ampicillin. A single colony for each construct was inoculated into LB broth and cultured at 37 °C with shaking (200 rpm). When the optical density at 600 nm (OD_600_) reached 0.5, dsRNA expression was induced by adding IPTG to a final concentration of 1 mM, followed by incubation for an additional 4 h. Bacterial cells were harvested by centrifugation, and dsRNA was extracted from the cell pellets using TRIzol^®^ Reagent according to the manufacturer’s instructions. The GFP sequence used for the control dsRNA corresponds to GenBank accession MN623123.1.

RNAi was performed using a dsRNA soaking method. For each treatment group (dsHc-NHR-49 or dsGFP control), three independent biological replicates were set up, with each replicate consisting of 5000 resistant larvae. The L3 were transferred to sterile 1.5 mL tubes and incubated with 100 μL of nuclease-free water containing 2000 ng/μL of the respective dsRNA. The tubes were incubated at 4 °C for 12 h to allow for dsRNA uptake. After incubation, the dsRNA solution was removed. Total RNA was then extracted from randomly selected larvae per replicate, reverse-transcribed into cDNA, and subjected to RT-qPCR to evaluate *Hc-NHR-49* knockdown efficiency.

### Motility assay

Following RNAi, the treated L3 larvae were collected, washed, and resuspended in sterile water. Approximately 20–50 larvae (in a 10 μL aliquot) were transferred to each well of a new 24-well cell culture plate. To each well, 1700 μL of sterile water and 90 μL of ivermectin stock solution were added to achieve a final concentration of 13.97 nmol/L. Control wells received an equivalent volume of 0.5% DMSO. The plates were incubated at 37 °C for 24 h. After incubation, larval movement was assessed by counting the number of head swings over a 1 min observation period for ten randomly selected larvae per well [28]. The entire experiment was performed with three independent biological replicates.

### Statistical analysis

All statistical analyses were performed using GraphPad Prism software (version 8.0.0 for Windows, GraphPad Software, USA). Data are presented as the mean ± standard error of the mean (SEM) from at least three independent biological replicates. Differences between two groups were analyzed using the non-parametric Mann–Whitney *U* test. A *P*-value of less than 0.05 was considered statistically significant.

## Results

### Amplification, cloning, and sequencing of *Hc-NHR-49*

**Fig. 1:**
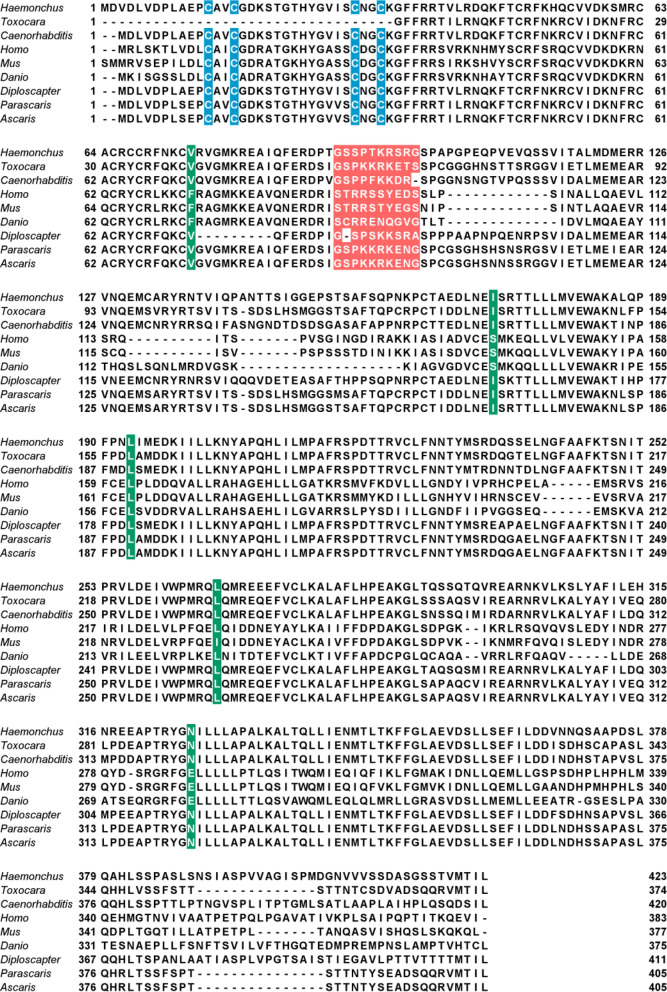
Alignment of the amino acid sequences of the NHR-49 from *Toxocara canis* (A0AOB2UP53), *Caenorhabditis elegans* (E5QCI8), *Homo sapiens* (P41235), *Mus musculus* (Q9WUU6), *Danio rerio* (Q8AXB6), *Diploscapter pachys* (A0A2A2K1B9), *Parascaris univalens* (A0A915BRC7), and *Ascaris suum* (F1KZG6). The C4-type steroid receptor zinc finger signature motif is highlighted in blue. The steroid hormone receptor signatures is highlighted in green. The nuclear localization signal is highlighted in pink

**Fig. 2:**
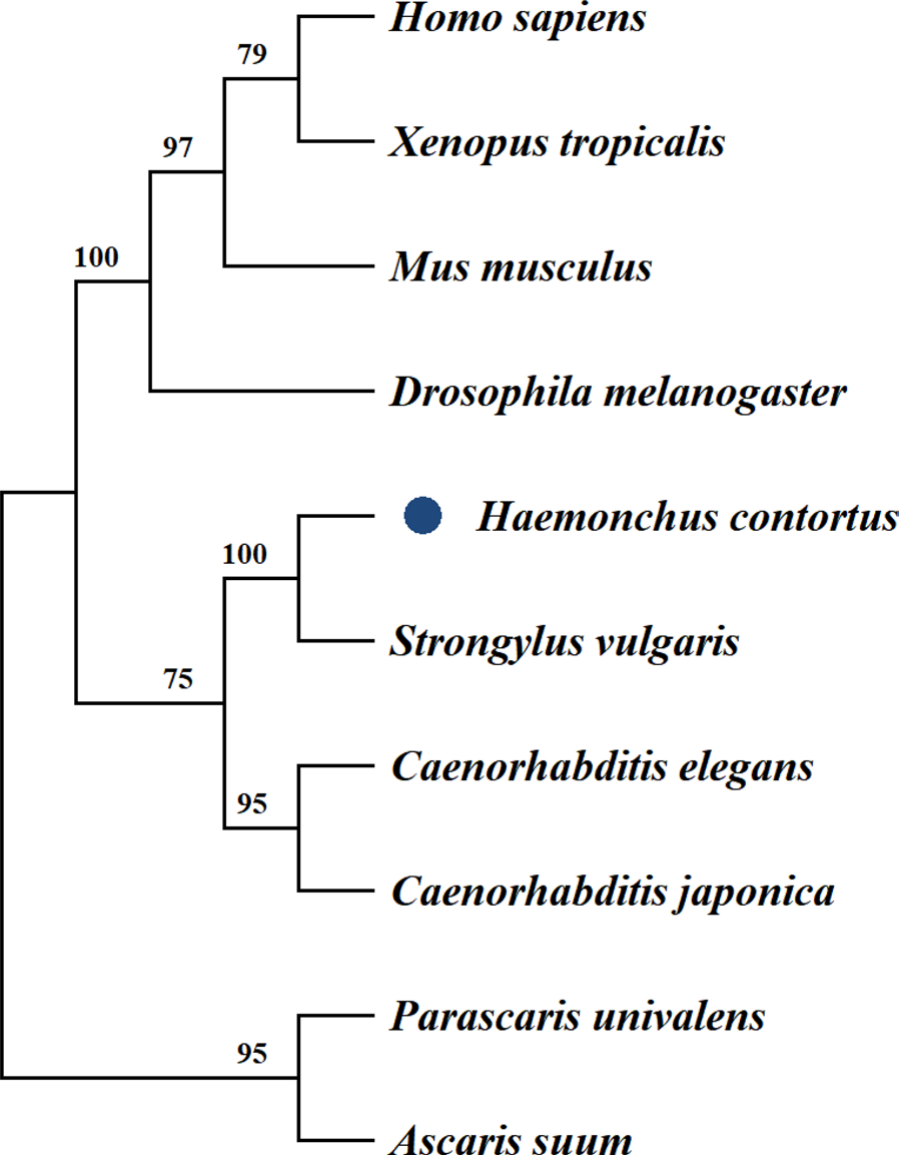
Neighbor-joining analysis of the *NHR-49* from *Caenorhabditis elegans* (E5QCI8), *Homo sapiens* (P41235), *Mus musculus* (Q9WUU6), *Parascaris univalens* (A0A915BRC7), *Ascaris suum* (F1KZG6), *Strongylus vulgaris* (A0A3P7IHR1), *Xenopus tropicalis* (A0A6I8QP06), *Caenorhabditis japonica* (A0A8R1E0I5), and *Drosophila melanogaster* (P49866). Evolutionary distances were computed using the Poisson correction method. Branch support values (1000 bootstraps) for nodes are indicated

The coding sequence of *Hc-NHR-49* comprised 1272 bp, encoding a protein of 423 amino acids with a theoretical molecular weight of 47 kDa and an isoelectric point (pI) of 7.87. No putative signal peptides or transmembrane domains were predicted by SignalP-5.0 and TMHMM-2.0 servers. A putative nuclear localization signal (NLS) motif, KRSR (residues 94–97), was identified within the sequence “GSSPTKRSRGS” (starting at position 91). A DNA-binding domain (residues 13–88) and a ligand-binding domain (residues 158–351) were identified (Fig. 1). The C4-type steroid receptor zinc finger signature motif (residues 13–33) and steroid hormone receptor signatures (residues 74–84, 172–193, 193–209, 266–281, and 326–343) were highly conserved. Phylogenetic analysis demonstrated that *Hc-NHR-49* shared high sequence identity with orthologs from *Caenorhabditis elegans* (82.4%) and *Caenorhabditis japonica* (83%) (Fig. 2).

### Transcriptional levels of *Hc-NHR-49* at different life stages in susceptible and resistant strains

**Fig. 3:**
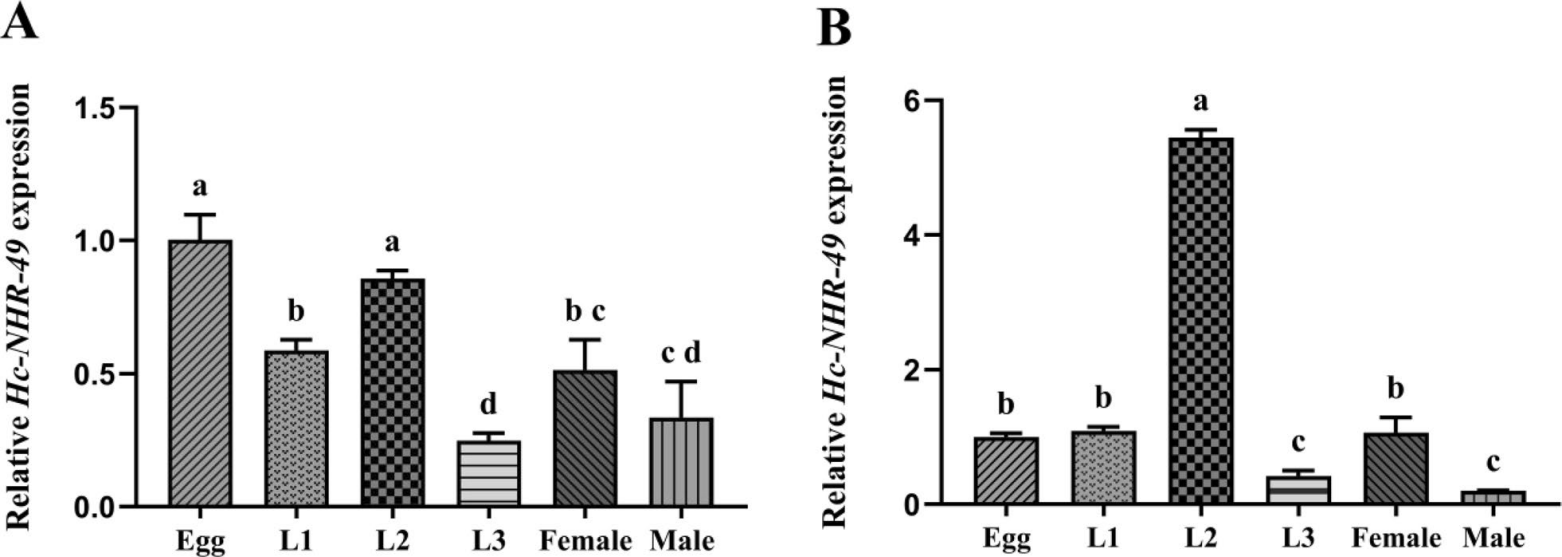
RT-qPCR expression analysis of the *Hc-NHR-49* gene in different life stages in susceptible and resistant strains. **A** Susceptible strains (YCHc-022). **B** Resistant strains (CYHHc-136). Error bars represent the standard error of the calculated mean based on three biological replicates. Different letters on the error bars show significant differences of the *Hc-NHR-49* gene among different developmental stages (*P* < 0.05)

The transcript of *Hc-NHR-49* was detected at all life stages (including eggs, L1, L2, L3, and adult males and females) in both susceptible and resistant strains of *H. contortus* using RT-qPCR. In the susceptible strains, the expression level of *Hc-NHR-49* was not significantly different in the L2 stage compared with eggs (Fig. 3a). However, it was significantly lower in other stages, with expression levels reduced to approximately 41% (*P* = 0.0003), 65% (*P* = 0.0001), 49% (*P* = 0.0001), and 66% (*P* = 0.0001) of those in eggs for the L1, L3, female, and male stages, respectively (Fig. 3a). In contrast, in the resistant strains, *Hc-NHR-49* expression was significantly upregulated (5.44-fold, *P* = 0.0001) only in the L2 stage relative to eggs (Fig. 3b). Conversely, it was significantly downregulated in the L3 and male stages, with expression levels 58% (*P* = 0.0002) and 80% (*P* = 0.0001) of those in eggs, respectively. No significant differences were observed in the remaining stages (L1 and female).

### Inductive effects of *Hc-NHR-49* under the treatment of ivermectin

**Fig. 4:**
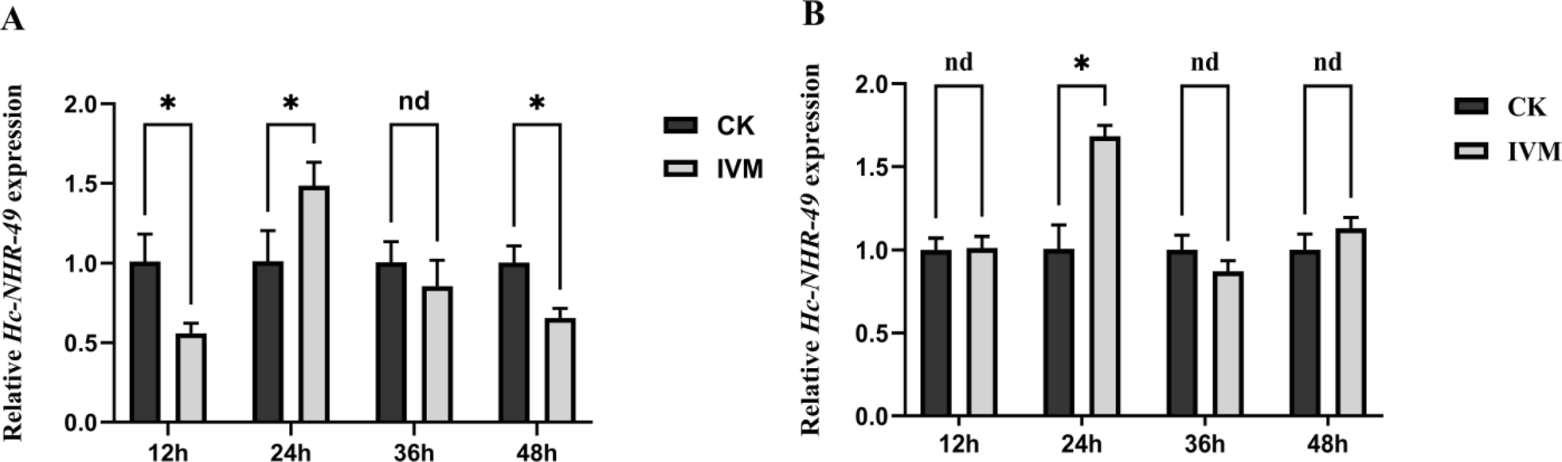
Relative expression levels of the *Hc-NHR-49* gene in L3 under the treatment of ivermectin. **A** EC_50_ treatment in susceptible strains (YCHc-022); **B** EC_50_ treatment in resistant strains (CYHHc-136). Error bars represent the standard error of the calculated mean based on three biological replicates. Different asterisks on the error bars show significant differences of the *Hc-NHR-49* gene between induction and control (^*^P < 0.05)

To further expore the relationship between the *Hc-NHR-49* and ivermectin resistance in *H. contortus*, ivermectin at EC_50_ was used to induce their expression in both susceptible and resistant strains. The expression levels of *Hc-NHR-49* after ivermectin exposure were detected at different times. In the susceptible strain (Fig. 4a), *Hc-NHR-49* expression showed a biphasic response. It was significantly downregulated at 12 h and 48 h (to 74% and 36% of the control level, respectively; both *P* = 0.007), but exhibited a transient 2.36-fold upregulation at 24 h (*P* = 0.009) before returning to baseline by 36 h. In contrast, the resistant strain (Fig. 4b) displayed a simpler pattern: no change at 12 h, followed by a significant 1.68-fold upregulation at 24 h (*P* = 0.006), and a return to control levels by 36 h.

### Recombinant *Hc-NHR-49* expression, purification, and polyclonal antibody detection

**Fig. 5:**
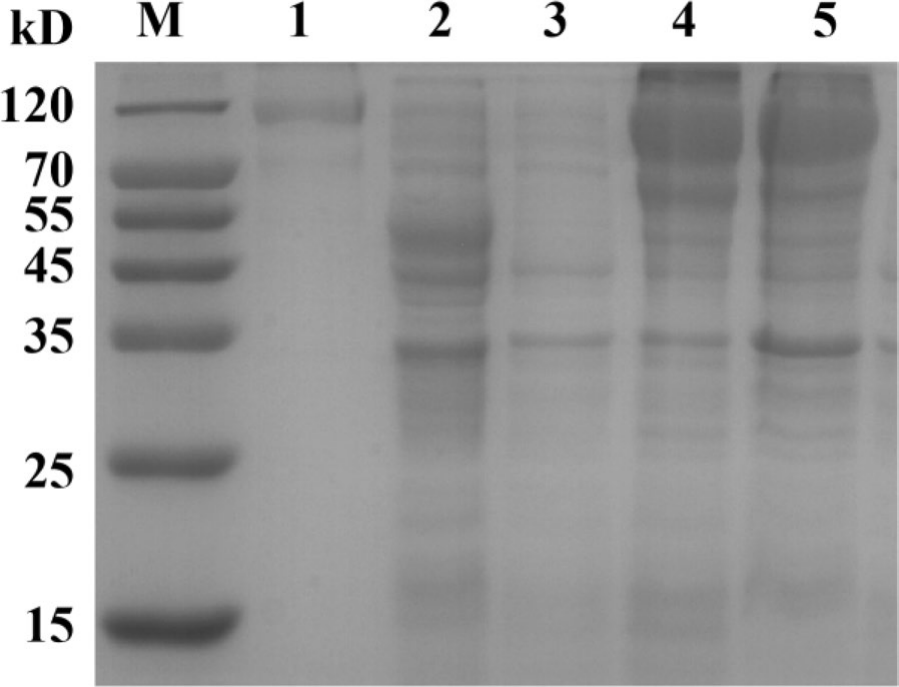
SDS-PAGE analysis of recombinant *Hc-NHR-49* from *H. contortus*. M: The molecular size marker. 1: Purified recombinant *Hc-NHR-49* protein. 2: *E.coli* lysate control (harboring only the pCold-TF expression vector). 3: Bacterial lysate of recombinant protein before induction. 4: Bacterial lysate supernatant of recombinant protein after induction by IPTG. 5: Bacterial lysate precipitation of recombinant protein after induction

**Fig. 6:**
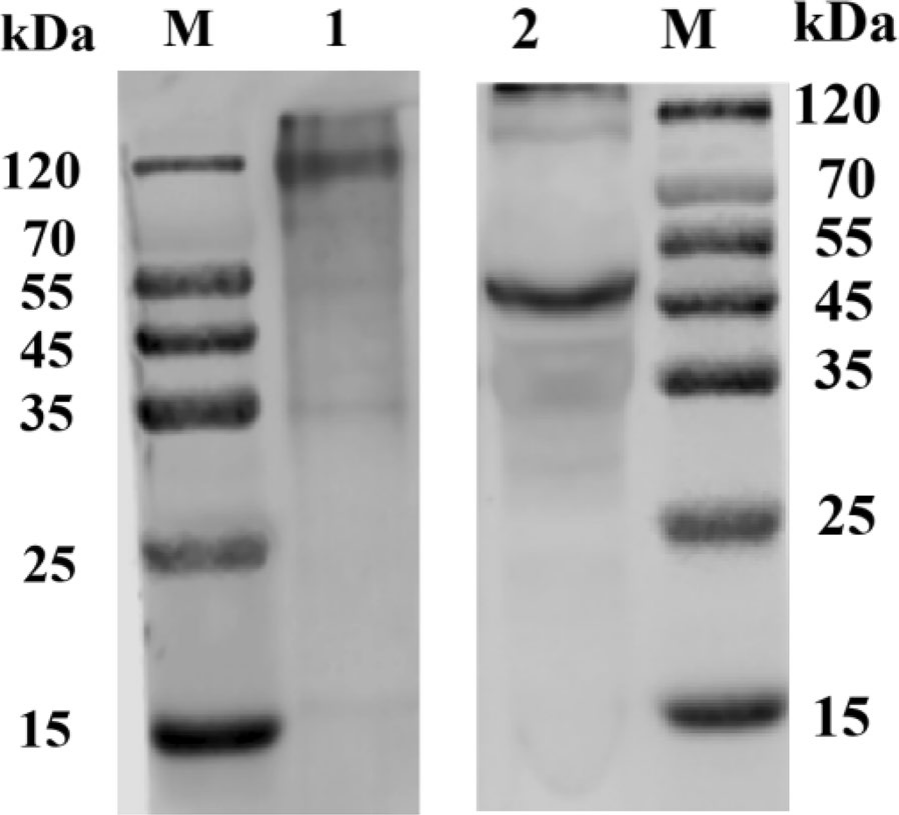
Western blot analysis of purified rHc-NHR-49 and soluble nematode extraction. Anti-rHc-NHR-49 serum recognized purified rHc-NHR-49 and native *Hc-NHR-49*. M: Prestained protein ladder. 1: Purified rHc-NHR-49 proteins were identified with mouse polyclonal anti-rHc-NHR-49 serum (1:500). 2: Natural *Hc-NHR-49* proteins of *H. contortus* were identified with mouse polyclonal anti-rHc-NHR-49 serum (1:500)

The recombinant *Hc-NHR-49* protein was expressed in *E. coli* BL21 (DE3) cells induced by IPTG. SDS-PAGE analysis of bacterial lysates revealed a predominant induced band at ~47 kDa, with the majority of the protein present in the soluble supernatant fraction (Fig. 5). This soluble fraction was subsequently subjected to Ni–NTA affinity chromatography, yielding a single purified band at the expected size (Fig. 5). To evaluate antibody specificity, Western blot analysis was performed using mouse anti-rHc-NHR-49 antiserum against both the purified recombinant protein and soluble somatic extracts from *H. contortus*. As shown in Fig. 6, the antiserum specifically recognized a single band corresponding to the recombinant protein and a native protein of comparable size in the nematode extracts. The observed molecular weight of rHc-NHR-49 (~96 kDa) exceeds its calculated weight based on the amino acid sequence, which is attributable to the 49 kDa fusion tag from the pCold-TF vector.

### Localization of *Hc-NHR-49* in *H. contortus*

**Fig. 7:**
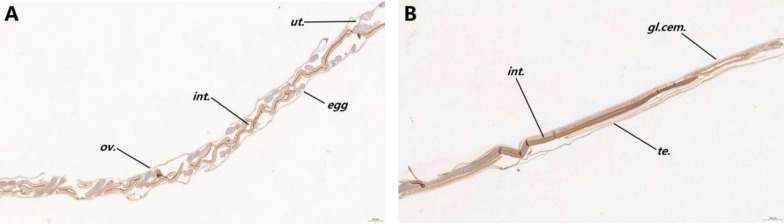
Immunohistochemical localization of *Hc-NHR-49* in female and male *H. contortus*. **A** Female, the uterus (ut), ovaries (ov), and intestine (int). **B** Male, the testes (te), cement gland (gl.cem), and intestine

To visualize the distribution of *Hc-NHR-49*, immunohistochemical staining was conducted on sections of both female and male *H. contortus*. Consecutive 5 μm sections were incubated with mouse anti-*Hc-NHR-49* serum. This revealed specific *Hc-NHR-49* expression in female reproductive organs—the uterus (ut) and ovaries (ov)—and the intestine (int) (Fig. 7a). In males, expression was localized to the testes (te), cement gland (gl.cem), and intestine (Fig. 7b). Notably, intestinal staining intensity was markedly higher in both sexes, suggesting abundant *Hc-NHR-49* protein in this tissue.

### Knockdown of *Hc-NHR-49* increases ivermectin toxicity

**Fig. 8:**
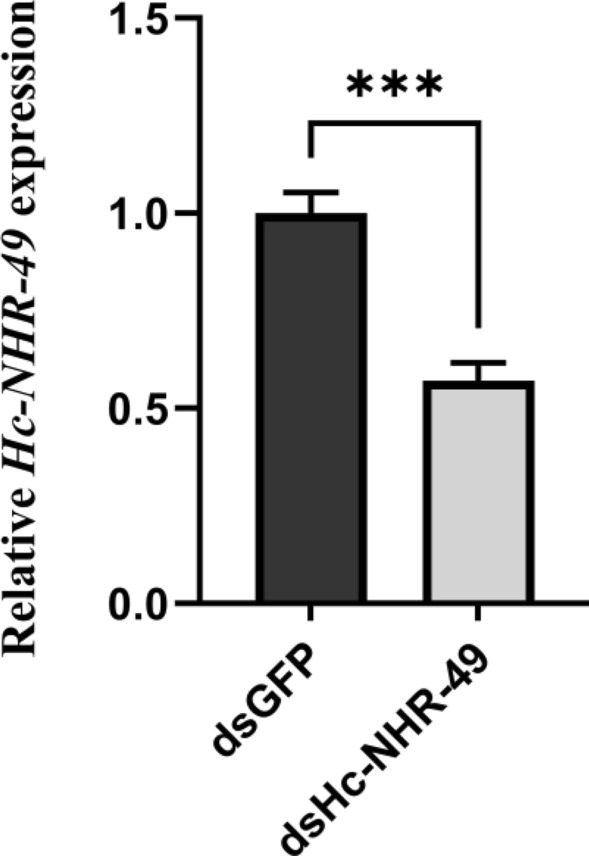
Expression of *Hc-NHR-49* gene determined by RT-qPCR in the resistant strains of L3 after RNAi. Error bars represent the standard error of the calculated means based on three biological replicates. Different asterisks on the error bars show significant differences of *Hc-NHR-49* gene between dsGFP and dsHc-NHR-49 groups (^***^P < 0.001)

**Fig. 9:**
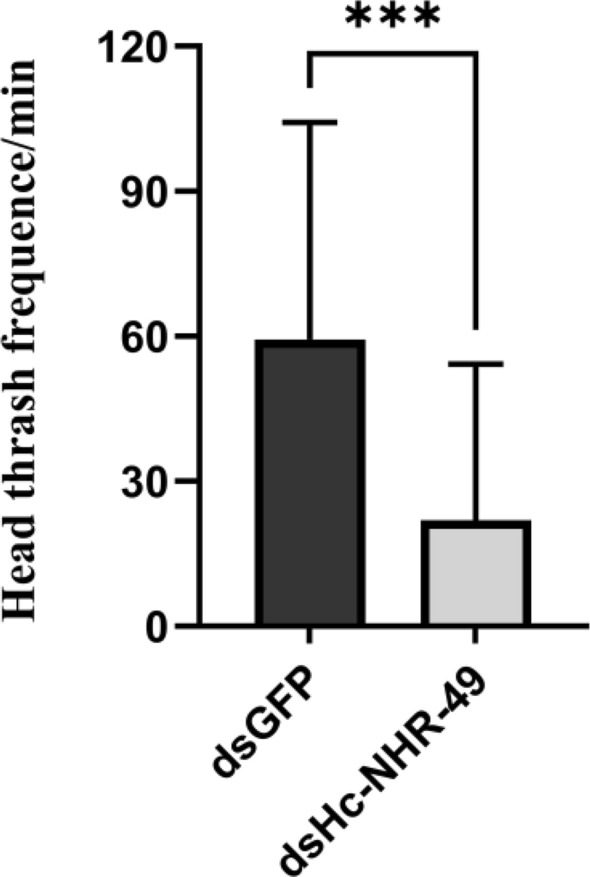
Head thrash frequence of the resistant *H. contortus* after silence of *Hc-NHR-49* and exploration to ivermectin at EC_50_. Error bars represent the standard errors of the calculated means based on three biological replicates. Different asterisks on the error bars show significant differences of head thrash frequence between dsGFP and dsHc-NHR-49 groups (^***^P < 0.001)

To investigate the functional role of *Hc-NHR-49* in ivermectin resistance, we knocked down its expression via RNAi in the resistant strain and assessed ivermectin toxicity. Treatment with 2000 ng/μL of dsHc-NHR-49 significantly reduced the expression of the target gene to 57% of the level in the dsGFP control group (*P* = 0.0001; Fig. 8). Consequently, when exposed to ivermectin at the EC_50_ concentration for the resistant strain, the head thrash frequency of the dsHc-NHR-49-treated worms was significantly reduced to 21.97/min, compared to 59.37/min in the dsGFP control group (*P* = 0.001; Fig. 9).

## Discussion

*Haemonchus contortus* a parasitic nematode that poses a significant threat to the global sheep industry. Control currently relies heavily on chemical anthelmintics, but the intensive use of ivermectin has led to widespread resistance, making the study of its underlying mechanisms crucial. This study investigates the role of the transcription factor *Hc-NHR-49* in ivermectin resistance, using a resistant (CYHHc-136) and a susceptible (YCHc-022) isolate. The resistant isolate has been maintained and passaged at our experimental station for 6 years without exposure to any anthelmintic drugs. Prior to the start of the experiments, we assessed its resistance level. The results showed that the EC_50_ was 13.97 μg/mL, with a resistance ratio of 10.04 (Fig S1; Table S2), confirming that the isolate has maintained a high level of ivermectin resistance. This finding is consistent with previous studies demonstrating that once anthelmintic resistance emerges in nematode populations, it can persist for extended periods, even in the absence of continued drug pressure. For instance, Egerton et al. passaged a resistant *H. contortus* isolate through sheep for approximately 2–3 years (11 generations) without further ivermectin exposure and observed no reversion to susceptibility [29]. The persistence of resistance in our isolate may be attributed to multiple factors, including the potential fitness cost of resistance being minimal or compensated, the fixation of resistance alleles in the population, and the involvement of complex genetic or epigenetic mechanisms that are not readily reversible [30].

Nuclear hormone receptors are ligand-modulated transcription factors that respond to steroids, fatty-acid-like molecules and other compounds [31]. Beyond its established roles, the *NHR-49* has emerged as a critical regulator of stress responses, directly modulating specific genes and pathways. However, the molecular and biochemical properties of *NHR-49* in the parasitic nematode *H. contortus* remain largely uncharacterized. In this study, we identified and characterized *NHR-49* in *H. contortus* for the first time. The cDNA was cloned, sequenced, and subjected to bioinformatic alignment with publicly available sequences from GenBank. Compared with *NHR-49* from *Homo sapiens*, *Mus musculus*, *Rattus norvegicus*, *Xenopus tropicalis*, and *C. elegans*, these domains are highly conserved. Phylogenetic analysis based on amino acid sequences showed that *Hc-NHR-49* is most closely related to that of *C. elegans* (82.4% sequence identity), while sequence identities with *Toxocara canis* and *Caenorhabditis japonica* were 85% and 83%, respectively, further confirming that NHR-49 is highly conserved across a variety of species. These results also suggest that *Hc-NHR-49* has similar functions to HNR-49 of these species.

The functions of NHR-49 are likely tissue-specific and may vary among species. In the model nematode *C. elegans*, somatic NHR-49 enables adaptation to physiological stress by up regulating genes involved in mitochondrial β-oxidation and fatty-acid desaturation [32]. In the parasitic nematode *H. contortus*, we found that *Hc-NHR-49* has a distinct and widespread tissue distribution, with strong expression in metabolically and reproductively active tissues—including the intestine, uterus, ovaries, testes, and cement gland. This ubiquitous expression pattern, contrasting with the more specific somatic role in *C. elegans*, indicates that *Hc-NHR-49* may fulfill multiple biological functions in the parasite, potentially extending beyond metabolic regulation.

In the model nematode *C. elegans*, *NHR-49* regulates mitochondrial/peroxisomal β-oxidation and fatty acid desaturation processes critical for development and adaptation to nutrient stress [33, 34]. *NHR-49* loss-of-function results in metabolic dysregulation, reduced lifespan and impaired survival under starvation [35]. Furthermore, it is required in specific germline stem cells for reproductive recovery after prolonged fasting [36]. Collectively, *NHR-49* emerges as a master regulator that promotes survival across diverse physiological contexts by reprogramming metabolism and restoring lipid homeostasis. Given this pivotal role in stress adaptation, we hypothesized that its ortholog in parasitic nematodes, *Hc-NHR-49*, might be involved in anthelmintic resistance. To test this, we analyzed *Hc-NHR-49* expression in *H. contortus*. We show that *Hc-NHR-49* expression varies throughout development in both ivermectin-susceptible and -resistant strains and, crucially, is dynamically regulated following ivermectin exposure. These findings extend the conserved role of *Hc-NHR-49* in stress responses to parasitic nematodes and specifically implicate *Hc-NHR-49* in the complex process of ivermectin resistance acquisition.

Although a previous genome-wide association study (GWAS) based on whole-genome resequencing identified *Hc-NHR-49* as a candidate gene for ivermectin resistance in *H. contortus* [19], its functional role remained unverified. Here, we employed RNAi to silence *Hc-NHR-49* in *H. contortus*. Following *Hc-NHR-49* knockdown and IVM treatment, a significant reduction in larval motility (head thrash frequency) was observed compared to controls. This finding indicates that *Hc-NHR-49* silencing enhances parasite sensitivity to IVM, thereby functionally validating its role as a key regulator of IVM susceptibility. This result aligns with the established functions of other nematode nuclear hormone receptors in anthelmintic response. For example, loss of *NHR-8* in *C. elegans* increases sensitivity to IVM [17], and *NHR-176* regulates the metabolism of anthelmintics in this model organism [37]. Furthermore, the same GWAS [19] implicated several other *Hc-NHR* genes (e.g., *Hc-NHR-3*, *Hc-NHR*−14, and *Hc-NHR*−22) in IVM detoxification. In contrast, IVM susceptibility is unaffected by the loss of *NHR-48* or *DAF-12* in *C. elegans* [38], highlighting the specificity of NHR functions. Collectively, our functional data establish *Hc-NHR-49* as a pivotal regulator of ivermectin resistance in *H. contortus*, where its targeted functional impairment re-sensitizes resistant parasites to the drug.

The overexpression of xenobiotic detoxification genes is a pleiotropic mechanism contributing to anthelmintic resistance in nematodes [39]. This includes the upregulation of ABC efflux transporters, which enhance anthelmintic elimination upon exposure to compounds such as ivermectin, thereby conferring resistance [40]. Consistently, detoxification metabolic enzymes are also overexpressed in IVM-resistant nematodes [8, 41, 42], with specific genes such as *GST-4* and *Pgp-9* implicated in ivermectin resistance in both *H. contortus* and *C. elegans* [43, 44]. The transcriptional regulation of these detoxification pathways is often mediated by nuclear hormone receptors. In *C. elegans*, for instance, *NHR-176* regulates *cyp-35d1*, a gene controlling the metabolism of thiabendazole [37]. Similarly, and of direct relevance to ivermectin resistance, deletion of *NHR-8* in nematodes significantly reduces the expression of key detoxification genes (including *Pgps*, *CYPs*, and *GSTs*) and increases susceptibility to ivermectin, demonstrating that NHR-8 is a master transcriptional regulator of detoxification and a determinant of anthelmintic sensitivity [45]. This regulatory paradigm mirrors the “xenosensor” function of nuclear receptors in mammals, which orchestrate detoxification gene expression in response to xenobiotics. Given the established role of NHRs in governing detoxification and anthelmintic resistance, and based on our expression and functional data, we propose that *Hc-NHR-49* confers ivermectin resistance in *H. contortus* by regulating the expression of detoxification enzyme genes. Therefore, which specific detoxification metabolic enzymes are involved in the metabolic resistance mechanism of *Haemonchus contortus* to ivermectin, and which of these enzymes are regulated by *Hc-NHR-49*? These questions warrant further in-depth investigation.

## Conclusions

In summary, this study provides the first functional characterization of *Hc-NHR-49* in *H. contortus*, demonstrating its involvement in ivermectin resistance. We characterized the gene and produced a specific polyclonal antibody, enabling the determination of its tissue-specific expression pattern. Transcriptional profiling revealed dynamic expression of *Hc-NHR-49* both during development and in response to ivermectin exposure. Most importantly, functional analysis using RNAi-mediated knockdown and a phenotypic motility assay demonstrated that *Hc-NHR-49* might contribute to ivermectin resistance.

## Supplementary Information

Supplementary Material 1.

## Data Availability

Data supporting the main conclusions of this study are included in the manuscript.

## Abbreviations

NHRs: Nuclear hormone receptors
BPW: Barber’s pole worm
AR: Anthelmintic resistance
IVM: Ivermectin
P-gps: P-glycoproteins
ABC: ATP-binding cassette
GST: Glutathione S-transferase
RNAi: RNA interference
YCHc-022: Susceptible population of *H. contortus*
CYHHc-136: Ivermectin-resistant population of *H. contortus*
L3: Third-stage larvae
L2: Second-stage larvae
L1: First-stage larvae
EC_50_: Half maximal effective concentration
RT-qPCR: Quantitative reverse transcription polymerase chain reaction
IPTG: Isopropyl β-d-1-thiogalactopyranoside
SDS-PAGE: Sodium dodecyl sulfate polyacrylamide gel electrophoresis
PVDF: Polyvinylidene difluoride
PBST: Phosphate-buffered saline with Tween^®^ 20
DAB: 3,3′-Diaminobenzidine
dsRNA: Double-stranded RNA
GFP: Green fluorescent protein
DMSO: Dimethyl sulfoxide
NLS: Nuclear localization signal
GWAS: Genome-wide association study
DAF: Decay-accelerating factor
CYPs: Cytochromes P450
