# Supplementary material for: Transcriptional profiling and functional characterization of the Hc-NHR-49 gene in ivermectin resistance of Haemonchus contortus
Source: Parasit Vectors. 2026 May 23;19:293. doi: 10.1186/s13071-026-07433-x (PMC13374340; doi:10.1186/s13071-026-07433-x)

Table S1 Primers of the *Hc-NHR-49* gene used for PCR and RT-qPCR in this study.

| Primer | Sequence | Experiment |
| --- | --- | --- |
| NHR-49-F | GCTCGGTACCCTCGAGATGGCATTAAGCAAAAAAAAAGT | Cloning of *Hc-NHR-49* |
| NHR-49-R | GCAGGTCGACAAGCTTTCATAATATTGTCATCACAGTGCT |  |
| qNHR-49-F | GGCAGTAGCCCAACAAAACG | *Hc-NHR-49* for RT-qPCR |
| qNHR-49-R | CTGATTCACCCGACGTTCCA |  |
| qGAPDH-49-F | GCAAAGACCCCGCTGAAATC |  |
| qGAPDH-49-R | TCGGCAGAAGGAGCAGAGAT |  |
| dsNHR-49-F | CGGGCCCCCCCTCGAGCGAGACCAATCAAGCGAACT | RNAi |
| dsNHR-49-R | ACCGCGGTGGCGGCCGCGTCGTCAAGGATGAACTCCGA |  |

Not: Restriction sites are underlined.

Table S2 Results of the larval development inhibition test. HC-S represents the international standard susceptible strain of *Haemonchus contortus*.

|  | EC_50_ (μg/mL) | RR | R^2^ | Level of Resistance |
| --- | --- | --- | --- | --- |
| Hc-S | 1.39 | - | 0.99 | - |
| CYHHc-136 | 13.97 | 10.04 | 0.94 | Resistant |
| YCHc-022 | 1.95 | 1.4 | 0.99 | Susceptible |

Not: RR:Resistance Ratio; R^2^: Coefficient of Determination; RR greater than 5 indicates the development of drug resistance.

Fig. S1 Results of the larval development inhibition test. CYHHc-136 (A); YCHc-022 (B); HC-S represents the international standard susceptible strain of *Haemonchus contortus*.


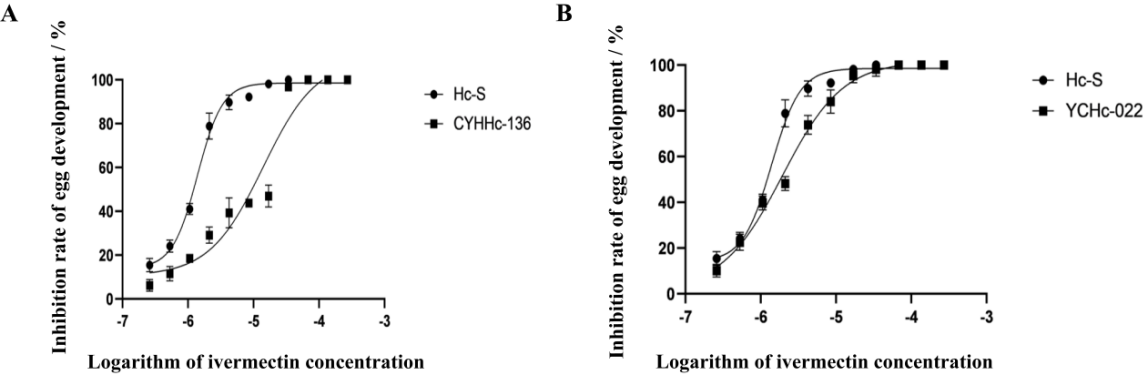

Supplement: Supplementary file 1 — Supplementary Material 1. [file 13071_2026_7433_MOESM1_ESM.docx]
